# Hepatotoxic potential of asarones: *in vitro* evaluation of hepatotoxicity and quantitative determination in herbal products

**DOI:** 10.3389/fphar.2015.00025

**Published:** 2015-02-20

**Authors:** Dhavalkumar N. Patel, Han K. Ho, Liesbet L. Tan, Mui-Mui B. Tan, Qian Zhang, Min-Yong Low, Cheng-Leng Chan, Hwee-Ling Koh

**Affiliations:** ^1^Department of Pharmacy, Faculty of Science, National University of Singapore, SingaporeSingapore; ^2^Health Products Regulation, Vigilance, Compliance and Enforcement Division, Health Sciences Authority, SingaporeSingapore; ^3^Applied Sciences Group, Pharmaceutical Division, Health Sciences Authority, SingaporeSingapore

**Keywords:** hepatotoxicity, drug induced liver injury, adverse events, herbal medicine, gas chromatography-mass spectrometry, asarones

## Abstract

α and β asarones are natural constituents of some aromatic plants, especially species of the genus *Acorus* (Araceae). In addition to reports of beneficial properties of asarones, genotoxicity and carcinogenicity are also reported. Due to potential toxic effects of β-asarone, a limit of exposure from herbal products of ~2 μg/kg body weight/day has been set temporarily until a full benefit/risk assessment has been carried out by the European Medicines Agency. Therefore, it is important to monitor levels of β-asarone in herbal products. In this study, we developed a simple, rapid and validated GC-MS method for quantitative determination of asarones and applied it in 20 pediatric herbal products after detecting high concentrations of β-asarone in a product suspected to be implicated in hepatotoxicity in a 3 month old infant. Furthermore, targeted toxicological effects were further investigated in human hepatocytes (THLE-2 cells) by employing various in *vitro* assays, with the goal of elucidating possible mechanisms for the observed toxicity. Results showed that some of the products contained as much as 4–25 times greater amounts of β-asarone than the recommended levels. In 4 of 10 samples found to contain asarones, the presence of asarones could not be linked to the labeled ingredients, possibly due to poor quality control. Cell-based investigations in THLE-2 cells confirmed the cytotoxicity of β-asarone (IC_50_ = 40.0 ± 2.0 μg/mL) which was associated with significant lipid peroxidation and glutathione depletion. This observed cytotoxic effect is likely due to induction of oxidative stress by asarones. Overall, the results of this study ascertained the usability of this GC-MS method for the quantitative determination of asarones from herbal products, and shed light on the importance of controlling the concentration of potentially toxic asarones in herbal products to safeguard consumer safety, especially when the target consumers are young children. Further investigations of the toxicity of asarones are warranted.

## Introduction

**Figure 1:**
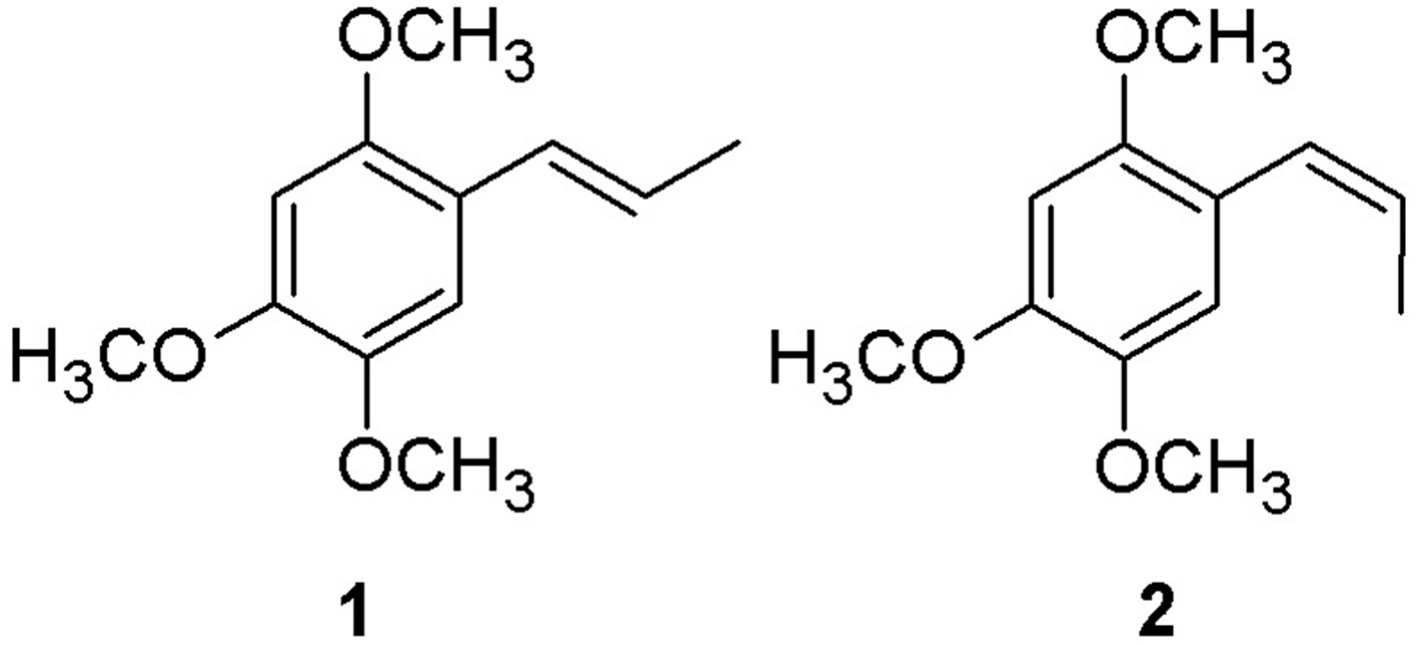
**Chemical structures of α-asarone (1) and β-asarone (2)**.

α-asarone (**1**) and β-asarone (**2**), the cis-trans isomers (Figure 1) are the natural constituents of some aromatic plants and their essential oil fractions, especially species of the genus *Acorus* (Araceae) including *Acorus calamus, Acorus tatarinowii* Schott and *Acorus gramineus* Soland (Commission of Chinese Pharmacopoeia, 2005; European Medicines Agency, 2005). *Acorus tatarinowii* Schott is referred to as *Acori tatarinowi* Rhizoma or shi chang pu and consists of the dried rhizome, cut in slices, of *Acorus tatarinowii* Schott (Commission of Chinese Pharmacopoeia, 2005). Asarones are reported as the major components of their essential oil (Commission of Chinese Pharmacopoeia, 2005; European Medicines Agency, 2005). Asarones are reported to possess several beneficial pharmacological properties (Lim et al., 2014; Meng et al., 2014; Wang et al., 2014). Specifically, β-asarone is reported to cross blood brain barrier and possesses beneficial effects on central nervous system and cardiovascular system (Wu et al., 2005; Liu et al., 2010; Meng et al., 2014). It has shown beneficial effects in experimental models of diseases such as Alzheimer's disease (Geng et al., 2010; Li et al., 2010, 2012; Wei et al., 2013; Meng et al., 2014), cerebral ischemia (Yang et al., 2013) and epilepsy (Fu et al., 2008). Separately, α-asarone is reported to possess anti-epileptic and hypolipidemic properties (Garduno et al., 1997; Chen et al., 2013). The *in vitro* and *in vivo* neuroprotective effects of α-asarone (Cho et al., 2002; Limón et al., 2009) and β-asarone (Li et al., 2010; Zou et al., 2011; Nandakumar et al., 2013) have also been reported. In addition, radioprotection and prevention of genotoxicity and hematopoetic injury by α-asarone is also reported (Sandeep and Nair, 2011).

On the other hand, α-asarone and β-asarone are reported to be genotoxic and hepatocarcinogenic in rodents. There are also reports of their mammalian toxicity and carcinogenicity (Hasheminejad and Caldwell, 1994; European Medicines Agency, 2005). It is reported that β-asarone is more toxic than α-asarone (European Medicines Agency, 2005). In human studies, exposure to asarones from the herb *Acorus calamus*, which contains high concentrations of asarones, caused prolonged vomiting in 7 consumers (Björnstad et al., 2009). Cytotoxic and genotoxic effects of asarones against HepG2 cells are also reported (Unger and Melzig, 2012). The authors have demonstrated that after metabolic activation, β-asarone at concentrations higher than 50 μg/mL was genotoxic as indicated by positive Ames test and sister chromatid exchange test (Unger and Melzig, 2012). Due to potential toxic effects of asarone, in particular, β-asarone, the European Council has limited the presence of β-asarone to a maximum of 1 mg/kg in alcoholic beverages and seasoning for snack food and 0.1 mg/kg in other food stuffs and beverages (European Medicines Agency (EMEA), 2002). As an extension of the regulation (limitation of the intake of β-asarone from food and alcoholic beverages), a limit of exposure from herbal medicinal products of ~115 μg/day, i.e. about 2 μg/kg bw/day has been instituted temporarily until a full benefit/risk assessment has been carried out (European Medicines Agency, 2005).

While genotoxicity and carcinogenicity of α and β-asarones have been reported, hepatotoxic effects are not very well studied. Several reports and reviews on the involvement of herbal medicine and herbal products in hepatotoxicity have been published (MacGregor et al., 1989; Kaplowitz, 1997; Stedman, 2002; Stickel et al., 2005; Navarro, 2009; Patel et al., 2012; Strader et al., 2012; Abdualmjid and Sergi, 2013; Teschke et al., 2013; Navarro and Lucena, 2014; Navarro et al., 2014). These reports raise concerns on hepatotoxicity associated with herbal medicine, and thereby draw our consideration for the effect of asarones found in herbal products. One prior study has described the cytotoxic effects of α and β-asarones in HepG2 cells (Unger and Melzig, 2012). This isolated reported awaits further validation using independent models and for possible mechanisms of their cytotoxic effects to be determined. Furthermore, HepG2 as a cancerous cell line may confound the interpretation of true cytotoxicity with anti-cancer effects. Unlike normal hepatocytes, it has limited drug metabolizing capacity and may not recapitulate cytotoxicities arising from metabolites. Hence, alternative cell models with competent P450 function such as THLE2 cells may be needed (Pfeifer et al., 1993; Datta et al., 2007; Greene et al., 2010; Saha et al., 2010a,b; Krajka-KuŸniak et al., 2012; Zimmerman et al., 2012). López et al. (1993) studied effects of micromolar concentrations of α-asarone on cultured rat hepatocytes over 2 weeks. α-asarone was found to be hepatotoxic by causing morphologic and ultrastructural alterations, triacylglycerol accumulation (fatty liver), and inhibition of protein synthesis and secretion in cultured rat hepatocytes. To the best of our knowledge, there is no report of hepatotoxicity evaluation of β-asarone in hepatocytes although it is more hepatocarcinogenic and genotoxic compared to α-asarone (European Medicines Agency, 2005). For this reason, β-asarone is the focus of this investigation.

In view of the reported toxic effects of asarones, it is important to develop analytical methods for their determination in herbal products. Asarones have been studied previously by thin layer chromatography (TLC), high performance liquid chromatography (HPLC), high performance thin layer chromatography (HPTLC), gas chromatography-mass spectrometry (GC-MS) and capillary electrophoresis methods (Oprean et al., 1998; Hanson et al., 2005; Widmer et al., 2005; Chen et al., 2009; Kumari et al., 2009; Meng et al., 2014; Wang et al., 2014). High Performance Liquid Chromatograhpy tandem mass spectrometry is also reported for analysis of β-asarone in rat plasma (Nandakumar et al., 2013). Among these methods, GC-MS is widely used for the analysis of asarones because of their intrinsic volatility and specificity (Wang et al., 2014). However, existing GC-MS methods require long analysis time (at least 18 min) for the determination of asarones (Oprean et al., 1998; Björnstad et al., 2009; Chen et al., 2009; Kumari et al., 2009; Meng et al., 2014; Wang et al., 2014). Therefore, we aim to develop an improved GC-MS method for rapid analysis of asarones in herbal medicine and products derived from herbal medicine.

The present study was conducted as a result of detection of high concentration of β-asarone in a baby product (from one batch) suspected to be implicated in drug induced liver injury in Singapore. The hepatotoxicity was marked by worsening acute liver failure in an infant. The objectives of this study were to perform hepatotoxicity assessment of potentially toxic asarones in human hepatocytes and to develop a simple, reliable and rapid GC-MS method for the quantitative determination of asarones in herbal products.

## Materials and methods

### Chemicals and reagents

The analytical grade (ACS) ethanol and methanol were purchased from the Tedia Company (Fairfield, OH, USA). The water was treated with Milli-Q water purification system (Millipore, Bedford, USA). Trichloroacetic acid (TCA), sodium dodecyl sulfate (SDS) malondialdehyde (MDA), diethyl maleate, thiobaribituric acid (TBA), butylated hydroxytoluene (BHT), sodium hydroxide (NaOH) and bovine serum albumin (BSA) were obtained from Sigma-Aldrich (Singapore). Reference standards of α-asarone, trans-trimethoxycinnamic acid and eugenol were obtained from Sigma-Aldrich (Singapore). β-asarone was obtained from Karl-Roth (Germany). Authentic herb *Acori tatarinowii* Rhizoma was obtained from the National Institute for the Control of Pharmaceutical and Biological Products (NICPBP), China.

### Samples

Nineteen herbal products labeled for their use in children were purchased from local herbal retail shops in Singapore. Product 20 was supplied by the Health Sciences Authority of Singapore (HSA). One product, Product 20 from one batch, was suspected to be implicated in drug induced liver injury marked by worsening acute liver failure in a 3-month old infant in Singapore. As the original patient sample of Product 20 was not enough to perform necessary studies, same product from a different batch was purchased. All of the above 20 products were not found to be adulterated with any prescription drugs and did not exceed the legal permissible limits of toxic heavy metals when analyzed by in-house adulterant screening methods.

### Preparation of samples and standards

Hundred microgram of product or powdered plant material was extracted using 15 mL of HPLC grade methanol by ultrasonication (230 V, Branson model 5510, Danbury, CT, USA) for 15 min. The extracts were filtered and the final volume is adjusted to 15 mL with methanol. One milliliter of this extract was then filtered with a 0.45 μm membrane filter for GC–MS analysis. Stock solutions of α and β-asarone were prepared at the concentrations of 1 mg/mL respectively in HPLC grade methanol and stored at 24°C in dark. Working solutions were diluted from the stock solutions with methanol. A structurally similar compound, eugenol was used as an internal standard. Stock solution of 1 mg/mL of internal standard was also prepared and stored in dark at 24°C.

### Gas chromatography-mass spectrometry (GC-MS)

Agilent gas chromatography-mass spectrometry (Agilent 7980 GC-MS) instrument was used. A DB-5MS column of film thickness 0.25 μm, length 30.0 m and diameter 0.25 mm was used with the carrier gas (helium) set at 1 mL/min. The initial oven temperature was set at 80°C. It was then increased to 300°C at 25°C/min. The final temperature of 300°C was held for 3.2 min. The total running time was 12 min. The injection volume was 1 μl using splitless mode. The data acquisition system was controlled by MS ChemStation. The MS was operated in the electron impact (EI) mode. MS data were recorded for 4.70–12 min. Full scan mass spectra were collected between 50 and 550 *amu* at 1.53 scan s^−1^. For SIM mode, ions with *m/z* of 208.1 were monitored for the quantification of asarones. Ions with *m/z* of 193.1 and 165.1 were used as qualifier ions for asarones. For the internal standard, eugenol, *m/z* 164.1 was monitored. The Wiley standard chemical MS library, NIST library and spectra of reference standards were used in the identification when scan mode used for analysis.

### GC-MS method validation

The mixed standard working solutions at each concentration were injected in triplicate. The calibration curves were constructed by plotting the ratio of peak areas of the asarone standards to the peak area of internal standard against the corresponding concentration. The LOD and LOQ of GC-MS method were determined and calculated as the analyte concentrations with signal/noise ratios of 3 and 10, respectively. Accuracy and precision of method were determined by analyzing quality control (QC) samples at three concentrations (low, mid and high), representing the entire calibration range for α and β-asarone. The intra-day (*n* = 6) and inter-day (*n* = 3) variations (intermediate precision) were evaluated by three replicate measurements of the QC samples in a single day and on three consecutive days, respectively. The recoveries of asarones were determined by the standard addition method. Mixed standard solutions of α and β-asarone at three different concentrations were added into a powder of *A. tatarinowii* herb before extraction and were then analyzed in triplicate. The unspiked sample solution was concurrently analyzed. Stability of the stock solution after 1 week was evaluated by comparing the determined concentrations of QC samples prepared from the aged stock solution to that of freshly prepared stock solution. Autosampler stability or post-preparative stability was also evaluated by re-injecting the processed QC samples (*n* = 3) kept at 24°C for 48 h.

### Cell culture

THLE-2 cells purchased from the American Type Culture Collection, ATCC (Manassas, VA, USA). They were grown in LHC-9 medium (Life technologies, California, USA) in a flasks pre-coated with collagen (2.9 mg/mL), fibronectin (1 mg/mL) and bovine serum albumin (1 mg/mL according to ATCC guidelines. ApoTox™ Triplex assay kit and GSH-Glo™ Glutathione assay kit were obtained from Promega (Madison, Wisconsin, USA).

### Measurement of cytotoxicity

Cytotoxicity was evaluated using the WST-1 (Sodium 5-(2, 4-disulfophenyl)-2-(4-iodophenyl)-3-(4-nitrophenyl)-2H tetrazolium inner salt) assay (Roche, Germany) according to the manufacturer's instructions. THLE-2 cells were suspended at optimum density (5 × 10^4^ cells/mL) in LHC-9 medium supplemented with 10% fetal bovine serum (FBS). 100 μl of this suspension was dispensed into 96- well plates. The cells were then incubated for 24 h at 37°C with 5% CO_2_. The medium was then discarded from the wells. Hundred microliter of pure components (up to 1.25 mM) were then added to the wells separately. The plates were then incubated for 72 h at 37°C with 5% CO_2_. The supernatant from all the wells was discarded. Then 100 μl of WST-1 solution (10% in media) was added into each well. The plates were then incubated for 1.5 h at 37°C with 5% CO_2_. After incubation, the absorbance was measured at 440 nm. Cell viability was expressed as a percentage of the control wells (*n* = 6). The entire experiment was triplicated. The final results obtained were expressed as a mean for three cell line passages and the standard deviations (SD) were also calculated. Statistical analysis was performed according to Student's *t*-test by one way analysis of variance. Significant difference was taken as *p* < 0.05. The IC_50_ values were determined by Calcusyn 3.0 software (Biosoft, Cambridge, UK). Each reported value was the mean ± SD from the 3 independent experiments.

### Thiobarbituric acid reactive substances (TBARS) assay

TBARS assay was applied to estimate lipid peroxidation. THLE-2 cells (2 × 10^5^ cells/well) were seeded into pre-coated 6-well plates. The cells were then incubated for 24 h at 37°C with 5% CO_2_. After 24 h of incubation at 37°C with 5% CO_2_, the media was discarded. Extracts of pure components and positive control (Diethyl maleate, 2.5 mM) dissolved in DMSO were then added at different concentrations (0–0.8 mM). The plates were then incubated for 24 h at 37°C with 5% CO_2_. The media was then discarded and the wells were washed with 500 μl of phosphate buffer saline (PBS) twice. The cells were then harvested for the preparation of cell lysate. Cells from 6-well plates were scraped into a 1.5 mL centrifuge tube containing 500 μl of 2.5% trichloroacetic acid (TCA) using cell scraper. The TCA solution containing cells was then centrifuged at 13,000 rpm for 5 min. After centrifugation, the supernatant was collected for estimation of malondialdehyde (MDA) formation and the cell pellets were stored at −20°C until analysis by Bradford assay for protein estimation. To 80 μl of cell lysate solution or appropriate MDA standards in 96 well plates, 10 μl of 1 mM butylated hydroxytoluene (BHT) in ethanol, 50 μl of 50% TCA in water and 75 μl of 1.3% TBA in 75 mM NaOH were added. The plate was then incubated for 45 min at 60°C and the reaction was stopped by addition of 10 μl of 20% SDS in water. The plate was then centrifuged at 2000 rpm for 10 min at 4°C. The supernatant was then transferred carefully to another micro plate and fluorescence was measured (Ex: 485 nm/Em: 535 nm). Nanomoles of MDA formed per mg of protein (determined using Bradford's assay) were then calculated using MDA standard calibration curve. For Bradford's assay, 2 mg/mL bovine serum albumin (BSA) solution was diluted with 0.05 N NaOH to prepare a series of concentrations in the range 0.125–1 mg/mL to plot the calibration curve. The cell pellets obtained from cell lysate were dissolved in 500 μl of 0.05 N NaOH. 5 μl of BSA standard, re-suspended protein pellets or 0.05 N NaOH blank is added to microtitre plate. 250 μl of Bradford 1× dye reagent was then added to above solution in plate. The plate was then read at 595 nm after 15 min of incubation at room temperature. The protein concentration was then determined from BSA standard calibration curve. Statistical analysis was performed according to student's *t*-test by one way analysis of variance. Significant difference was taken as *p* < 0.05. Each reported value was the mean ± SD from 3 independent experiments.

### Glutathione depletion assay

This assay was performed by using GSH-Glo™ Glutathione assay kit from Promega (Madison, Wisconsin, USA). The kit contain total Glutathione (GSH) lysis reagent, oxidized Glutathione (GSSG) lysis reagent, luciferin generation reagent and glutathione standard. THLE-2 cells (10 × 10^3^ cells/well) were seeded into pre-coated 96-well plates. The cells were then incubated for 24 h at 37°C with 5% CO_2_. After 24 h, the medium was discarded and two sets of pure components dissolved in DMSO were then added at different concentrations in serum free media (one set for reduced glutathione (GSH) measurement and second set for oxidized glutathione (GSSG) measurement). Appropriate concentrations of glutathione standards were also added to wells without cells to obtain the glutathione calibration curve. The plates were then incubated for 72 h at 37°C with 5% CO_2_. The medium was then discarded from the wells and the cells were treated with glutathione measurement kit in accordance to the manufacturer instructions. Briefly, after removal of media 50 μl/well of total Glutathione (GSH) lysis reagent or Oxidized Glutathione (GSSG) lysis reagent, as appropriate for desired endpoint was added. To the wells containing glutathione standards, 50 μl/well of total Glutathione (GSH) lysis reagent was added. The plate was then shaken at room temperature for 5 min. Then 50 μl/well of luciferin generation reagent was added to all wells. The plate was then shaken briefly and incubated at room temperature for 30 min. After incubation, 100 μl/well of luciferin detection reagent was added. The plate was then shaken briefly and incubated at room temperature 15 min before measuring the luminescence using GloMax® Multi+ luminometer from Promega (Wisconsin, USA). Calibration curves for GSH and GSSG were then plotted (Note: Two moles of GSH are generated per 1 mole of GSSG. Dividing the GSH concentrations by two gives the concentration of GSSG). GSH and GSSG in THLE-2 cells after treatment were then calculated and the ratio of GSH/GSSG was calculated by following formula: Ratio GSH/GSSG treated = [μM GSH–(μM GSSG × 2)]/μM GSSG. Statistical analysis was performed according to student's *t*-test by one way analysis of variance. Significant difference was taken as *p* < 0.05. Each reported value was the mean ± SD from 3 independent experiments.

### Simultaneous measurement of cell viability and caspase-3/7 levels

ApoTox™ Triplex assay kit from Promega was used for the simultaneous measurement of caspase-3/7 activation and cell viability in the same sample well. The kit contains GF-AFC Substrate (for cell viability), bis-AAF-R110 Substrate (for cytotoxicity) and luminogenic DEVD-peptide substrate for caspase-3/7 and Ultra-Glo™ Recombinant Thermostable Luciferase (to measure caspase-3/7 activation). THLE-2 cells (10 × 10^3^ cells/well) were seeded into 96-well plates with white background. The cells were then incubated for 24 h at 37°C with 5% CO_2_. After incubation, extracts and pure compounds were then added at different concentrations in media to the wells containing cells for two different incubation periods (6 and 12 h). After incubation, 20 μl each of GF-AFC substrate (for cell viability) and bis-AAF-R110 substrate (for cytotoxicity) reagent were added to all wells, and briefly mixed by orbital shaking (300–500 rpm for ~30 s). The plates were then incubated at 37°C for 30 min. Then the fluorescence was measured at excitation wavelength of 400 nm and emission wavelength of 405 nm to measure cell viability. To the same plate, 100 μl of Caspase-Glo® 3/7 reagent was added to all wells, and briefly mixed by orbital shaking (300–500 rpm for ~30 s). The plates were then incubated for 30 min at room temperature before measuring luminescence (caspase activation, a hallmark of apoptosis). Average percentage cell viability and caspase-3/7 activation for a well was expressed as a percentage of the control wells by comparing the average fluorescence and luminescence of treated wells against that of control wells in a triplicate manner. The results obtained were expressed as a mean ± SD for three cell line passages.

## Results

### Method development and validation

Steam distillation and /or hydrodistillation are frequently used methods for determination of β-asarone in plant materials (Oprean et al., 1998; Kumari et al., 2009). However, such distillation methods require long extraction time and have more processing steps. Ultrasonication is a very powerful and efficient method for the extraction of plant material and does not involve heat, which may degrade some of the thermally unstable products. Therefore, ultrasonication method was chosen instead of other methods such as Soxhlet extraction, maceration and steam distillation. Therefore, in this study, ultrasonication method was optimized for the extraction of asarones from botanical health products.

**Figure 2:**
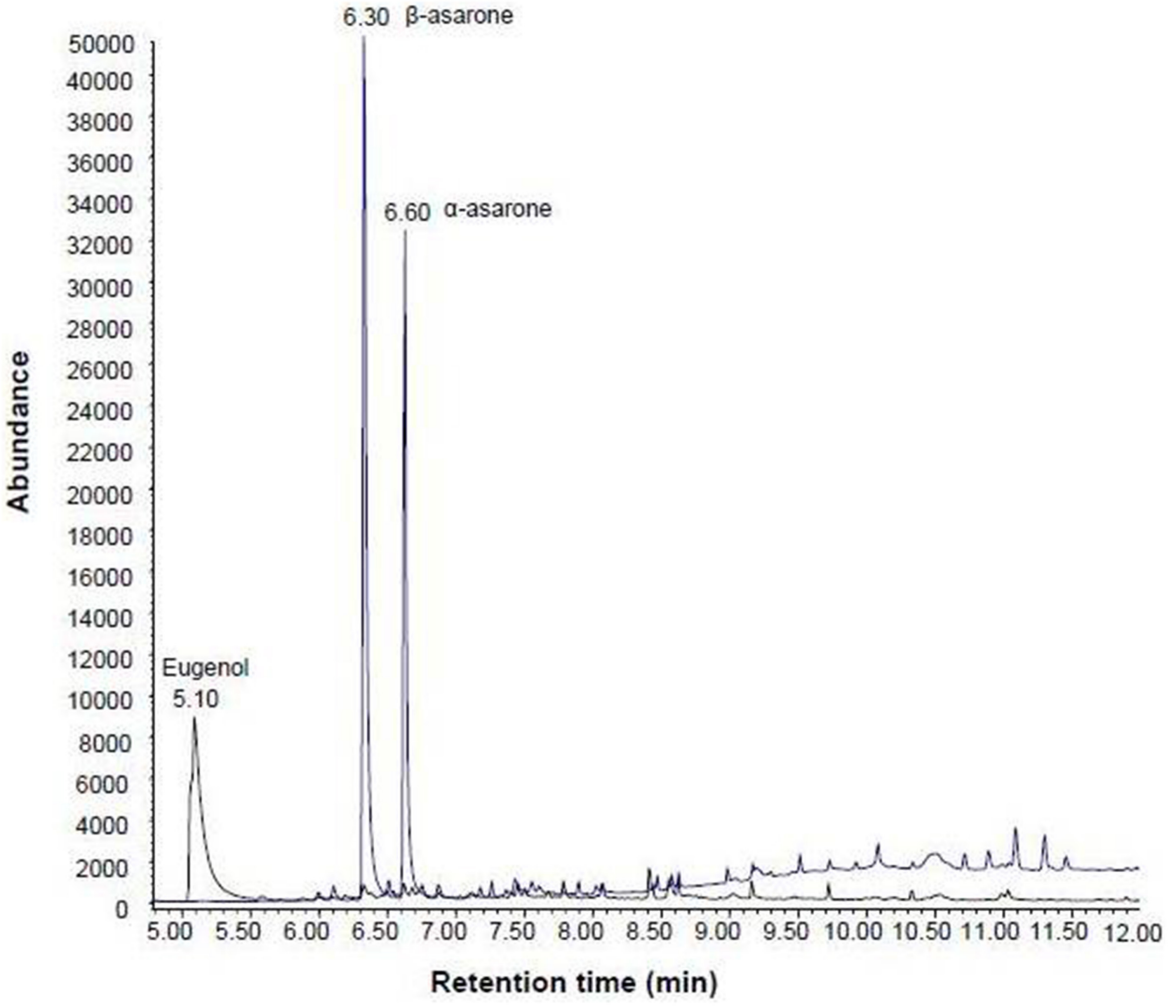
**GC-MS chromatograms of Product 18**. The internal standard eugenol, β-asarone and α-asarone were well separated at the retention time of 5.1, 6.3, and 6.6 min respectively.

The GC-MS method was shortened from initial screening method of 30 min to 12 min for rapid quantification of asarones. This was achieved by optimizing temperature ramp to separate α-asarone, β-asarone and the internal standard eugenol. Although β and α-asarones are structural isomers, they were well resolved at 6.3 and 6.6 min respectively (Figure 2). Eugenol eluted at 5.1 min (Figure 2). Mass spectra of β-asarone, α-asarone and eugenol were initially obtained by running in scan mode. From these mass spectra, ions with *m/z* of 208.1 and 164.1 were selected in SIM mode for asarones and eugenol respectively for quantification. This GC-MS method has been validated. The linearity data, concentration range, LOD and LOQ are presented in Table 1. Absolute recovery or the extraction efficiency of asarones (Table 2) was studied at three different concentrations from the *A. tatarinowii* herb. Data of analytical recoveries of QC samples for asarones and the stability of asarones at the tested conditions are presented as supplementary materials (Tables S1, S2 respectively). The calibration curves were found to have good linearity (*R*^2^ > 0.999). The RSDs of intra- and inter-day precision for α and β-asarones were 4.31–7.69 and 2.59–6.61 respectively. The accuracy of the developed method was found to be satisfactory as the percentage deviations of the QC samples from the nominal concentration were all within the acceptable limit of ± 10% for the recovery of asarones. The absolute recoveries or extraction efficiency of asarones were all above 90%. The results of stability studies under different conditions have shown that asarones were stable at the tested storage and handling conditions.

**Table 1:** **Linear regression data for the GC-MS method for quantification of asarones**.

**No**.	**Asarone**	**Calibration curve**	***R*^2^**	**Linear range (ng/mL)**	**LOD (ng/mL)**	**LOQ (ng/mL)**
1	β-asarone	*y* = 0.0014x + 0.1248	0.9993	156–5000	4.2	12.6
2	α-asarone	*y* = 0.0016x + 0.2398	0.9997	156–5000	4.6	13.9

**Table 2:** **Absolute recoveries or extraction efficiency of asarones from the herb *A. tatarinowii***.

**Asarone**	**Concentration of QC sample (ng/mL)**	**Mean recovery**			
		**Intra-day (*n* = *6*)**		**Inter-day (*n* = *9*)**	
		**% recovery ± SD**	**% RSD**	**% recovery ± SD**	**% RSD**
β-asarone	400	94.65±4.67	4.94	95.75±5.33	5.57
	1000	92.84±5.89	6.34	93.79±7.21	7.69
	4000	96.02±4.15	4.32	96.59±4.87	5.50
α-asarone	400	96.61±6.02	6.23	94.45±6.24	6.61
	1000	91.46±3.15	3.44	90.61±2.35	2.59
	4000	102.36±4.22	4.12	101.03±4.56	4.51

### Quantification of asarones in herbal products

As discussed earlier, asarones are potentially toxic compounds and therefore their concentrations in herbal medicinal products particularly products used for infants and children who are more prone to adverse events should be monitored. Hence, we conducted a study to explore the prevalence of asarones in baby products marketed in Singapore. Table 3 shows the analysis of 20 products for the presence of asarones. Of the 20 samples analyzed, α and β asarones were found to be present in 10 products (50%). In six of 10 samples, the presence of asarone could be attributed to the labeled ingredients. However, in four products, the presence of asarones could not be explained by any of the ingredients as labeled. This could be attributed to poor quality control measure and/or mixing of herbs during manufacturing. Subsequently, asarones were quantified in 10 products found to contain asarones by the validated GC-MS method. In addition, concentrations of asarones in the authentic herb *Acori tatarinowii* were also estimated. The herb was found to contain 7679.8 ± 85.7 mg/kg of β-asarone and 679.1 ± 20.3 mg/kg of α-asarone. All 10 products were found to contain more than the recommended amount of β-asarone as stipulated by EMEA (1 mg/kg for alcoholic beverages and 0.1 mg/kg for other food stuffs). Particularly, Product 20 was found to contain highest amount of β-asarone from the 10 products. Table 4 also shows the estimated exposure of β-asarones to the consumers of baby products that were found to contain asarones. This estimation was based on the average body weights of normal healthy consumers in Singapore (Health Promotion Board, Singapore). The quantification and estimation of exposure of asarones indicates that consumers are likely to get exposed to the levels of β-asarones well above the recommended exposure levels. Of the 10 products found to contain β-asarone, 6 products were found to contain β-asarone more than the daily recommended exposure levels. These products were found to contain as much as 4–25 times more amount of β-asarone than the recommended amount. These results suggest that the β-asarone (in varied concentrations) is prevalent in commercial herbal products available in the local market meant for children and infants.

**Table 3:** **Screening and quantification of β-asarone and α asarone in herbal products for children by GC-MS**.

**No**.	**Name**	**Concentration of β-asarone (mg/kg)**	**Concentration of α-asarone (mg/kg)**	**Source**
1	Product 1	Not detected	Not detected	–
2	Product 2	Not detected	Not detected	–
3	Product 3	273.6 ± 21.6	40 ± 1.2	*Acori tatarinowii* Rhizoma
4	Product 4	Not detected	Not detected	–
5	Product 5	230.9 ± 21.3	34.7 ± 1.2	*Acori tatarinowii* Rhizoma
6	Product 6	4.8 ± 0.2	5.0 ± 0.2	*Acori tatarinowii* Rhizoma
7	Product 7	11.2 ± 0.1	47.7 ± 3.0	*Acori graminei* Rhizoma
8	Product 8	181.6 ± 5.4	106.1 ± 1.0	–
9	Product 9	Not detected	Not detected	–
10	Product 10	Not detected	Not detected	–
11	Product 11	181.6 ± 5.4	106.1 ± 1.0	unknown source
12	Product 12	4.3 ± 0.3	13.3 ± 0.1	unknown source
13	Product 13	3.2 ± 0.2	10.9 ± 3.3	*Asari radix et* Rhizoma
14	Product 14	241.5 ± 10.5	53.2 ± 1.2	unknown source
15	Product 15	Not detected	Not detected	–
16	Product 16	Not detected	Not detected	–
17	Product 17	Not detected	Not detected	–
18	Product 18	243.5 ± 18.2	144.3 ± 7.1	unknown source
19	Product 19	Not detected	Not detected	–
20	Product 20	425.7 ± 28.2	62.8 ± 3.1	*Acori tatarinowii* Rhizoma
21	Authentic herb A*cori tatarinowii*	7679.8 ± 85.7	679.1 ± 20.3	*Acori tatarinowii* Rhizoma

**Table 4:** **Estimation of daily exposure levels of β-asarone in herbal products for children**.

**Product**	**Dose**	**Daily exposure of β-asarone based on quantification (μg/day)**	**Recommended daily allowance as per EMEA guidelines (μg/day)**
Product 3	Infants 6 months to 1 year: ½tsp; 1–3 years: 1 tsp; 3–6 years: 1–2 tsp; Adults: one bottle each time. 3 times a day.	≤ 1 year: 172	≤ 1 year: 17–22
		1–3 years: 344	1–3 years: 22–27
		3–6 years: 344–688	3–6 years: 41
		Adults: 1067	Adults: 115
Product 5	1–6 years: ½tsp flat; 7–12 years: 1 tsp flat; Adults:1/2 bottle. 3 times daily	1–6 years: 180	1–6 years: 22–41
		7–12 years: 360	7–12 years: 41–72
		Adults: 2880	Adults: 115
Product 6	1–6 years: ½tsp flat; 7–12 years: 1 tsp flat; Adults:1/2 bottle. 3 times daily	1–6 years: 4	1–6 years: 22–41
		7–12 years: 8	7–12 years: 41–72
		Adults: 58	Adults: 115
Product 7	1–3 years: 1 tsp each time; 3–6 years:1 or 2 tsp each time; adults:1 bottle each time, 3 times a day	1–3 years: 21	1–3 years: 22–27
		3–6 years: 21–42	3–6 years: 41
		Adults: 84	Adults: 115
Product 11	1–5 years: 1 tsp each time; 5 year or more; 1–2 tsp each time. 4 times a day	1–5 years: 370	1–5 years: 22–41
		≥ 5 years: ≥ 370	≥5 years: 41–115
Product 12	1–3 years: 400 mg each time; adults: 650 mg (1 bottle) each time. 2–3 times a day	1–3 years: 3.5	1–3 years: 22–27
		Adults: 5.6–8.4	Adults: 115
Product 13	One bottle each time, 2 times daily; infant mth old ½bottle each time, 2 times a day	Infants: 6.4	Infants: 14
		Adults: 12.8	Adults: 115
Product 14	3 months-1 year: ½bottles each time; children 1–5 years old: 1 bottle each time; Children 5 years old and above: 1–2 bottles each time, 4 times a day.	≤ 1 year: 193	≤ 1 yr: 14–22
		1–5 years: 386	1–5 years: 22–41
		>5 years: 386–773	>5 years: 52–115
Product 18	1–5 years: 1 tsp each time; 5 years and above: 1–2 tsp each time. Four times a day.	1–5 years: 623	1–5 years: 22–41
		>5 years: >623	>5 years: 52–115
Product 20	1–3 years: one tube per day; 4–10 years: 2 tubes per day.	1–3 years: 128	1–3 years: 22–41
		4–10 years: 255	4–10 years: 41–72

### Determination of cell viability

The effects of asarones and extract of Product 20 on the cell viability of human hepatocytes were evaluated using THLE-2 cells. Treatment of THLE-2 cells with varied concentrations of α-asarone, β-asarone and extract of Product 20 for 72 h shows that asarones and extract of Product 20 reduces cell viability of hepatocytes in a dose dependent manner. The dose dependent effects of asarones and Product 20 were further evaluated to determine their IC_50_ values. The IC_50_values of α-asarone, β-asarone and Product 20 were found to be 46.0 ± 1.0 μg/mL (0.221 ± 0.001 mM), 40.0 ± 2.0 μg/mL (0.193 ± 0.001 mM), and 50.0 ± 0.8 μg/mL respectively. We have further evaluated effects of trans-trimethoxycinnamic acid, major metabolite of asarone (Björnstad et al., 2009) on hepatocytes. Compared to α and β-asarone, trans-trimethoxycinnamic acid was found to be very less toxic. The IC_50_ value could not be determined as it did not kill more than 50% cells even at highest concentration tested (i.e., IC_50_ > 350 μg/mL).

### TBARS assay to measure lipid peroxidation

**Figure 3:**
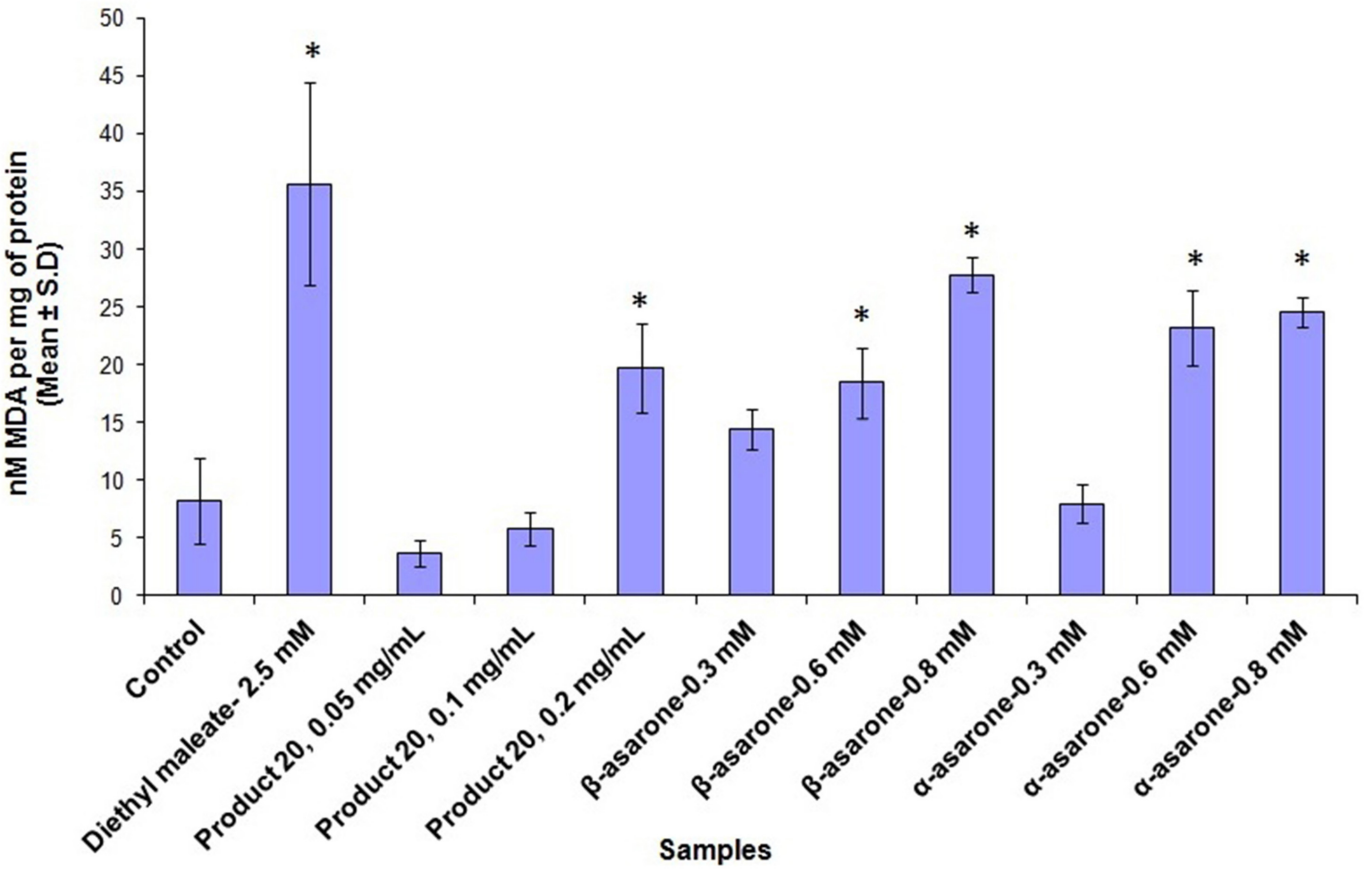
**The plot shows the average nanomoles of MDA per mg of protein ± standard deviation of three independent experiments upon treatment with methanol extracts of Product 20, α and β-asarones against THLE-2 cells for 24 h**. Significant difference (^*^) was taken as *p* < 0.05 in comparison with untreated control cells.

Determination of lipid peroxidation was performed using THLE-2 cells after 24 h of incubation using TBARS assay. Lipid peroxidation is quantified by measuring malondialdehyde (MDA), a breakdown product formed from polyunsaturated fatty acids (PUFA) hydroperoxides. Effects of asarones and extract of Product 20 on formation of MDA were determined at three different concentrations. The levels of MDA were increased with increasing concentration of asarones from 0.3 to 0.8 mM, as compared to vehicle control (Figure 3). The methanol extract of Product 20 was also found to have significant lipid peroxidation inducing effects at 0.2 mg/mL concentration. The asarones were also found to have cause similar level of lipid peroxidation as that of positive control diethyl maleate (2.5 mM) at 0.8 mM concentration.

### Glutathione depletion assay

**Figure 4:**
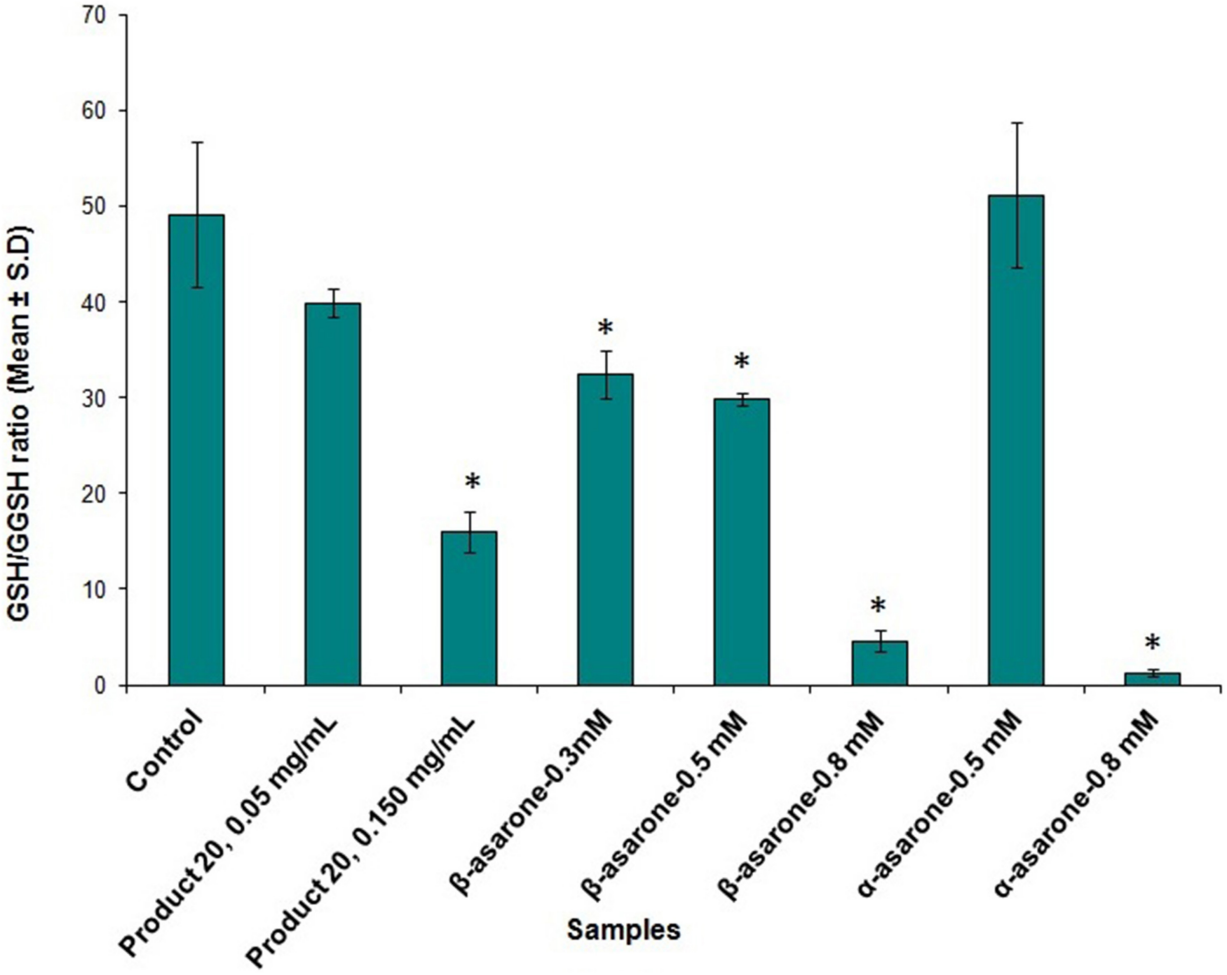
**The plot shows the ratio of GSH (reduced form)/GSSG (oxidized form) (mean ± SD, *n* = 3) upon treatment with methanol extracts of Product 20, compounds α and β-asarones against THLE-2 cells for 72 h**. Significant difference (^*^) was taken as *p* < 0.05 in comparison with untreated control cells.

Ratio of reduced glutathione (GSH) and oxidized glutathione (GSSG) was measured in THLE-2 cells after 72 h of incubation after treatment with asarones and Product 20. Increased levels of oxidized form of glutathione (formed as results of ROS scavenging) are indicative of oxidative stress. As can be seen from Figure 4, asarones produced significant glutathione depletion as evident by the increased formation of GSSG particularly at the concentration of 0.75 mM compared to vehicle control. Similarly, methanol extract of Product 20 also depleted GSH levels at 0.15 mg/mL concentration.

### Simultaneous measurement of cell viability and caspase-3/7 levels

**Figure 5:**
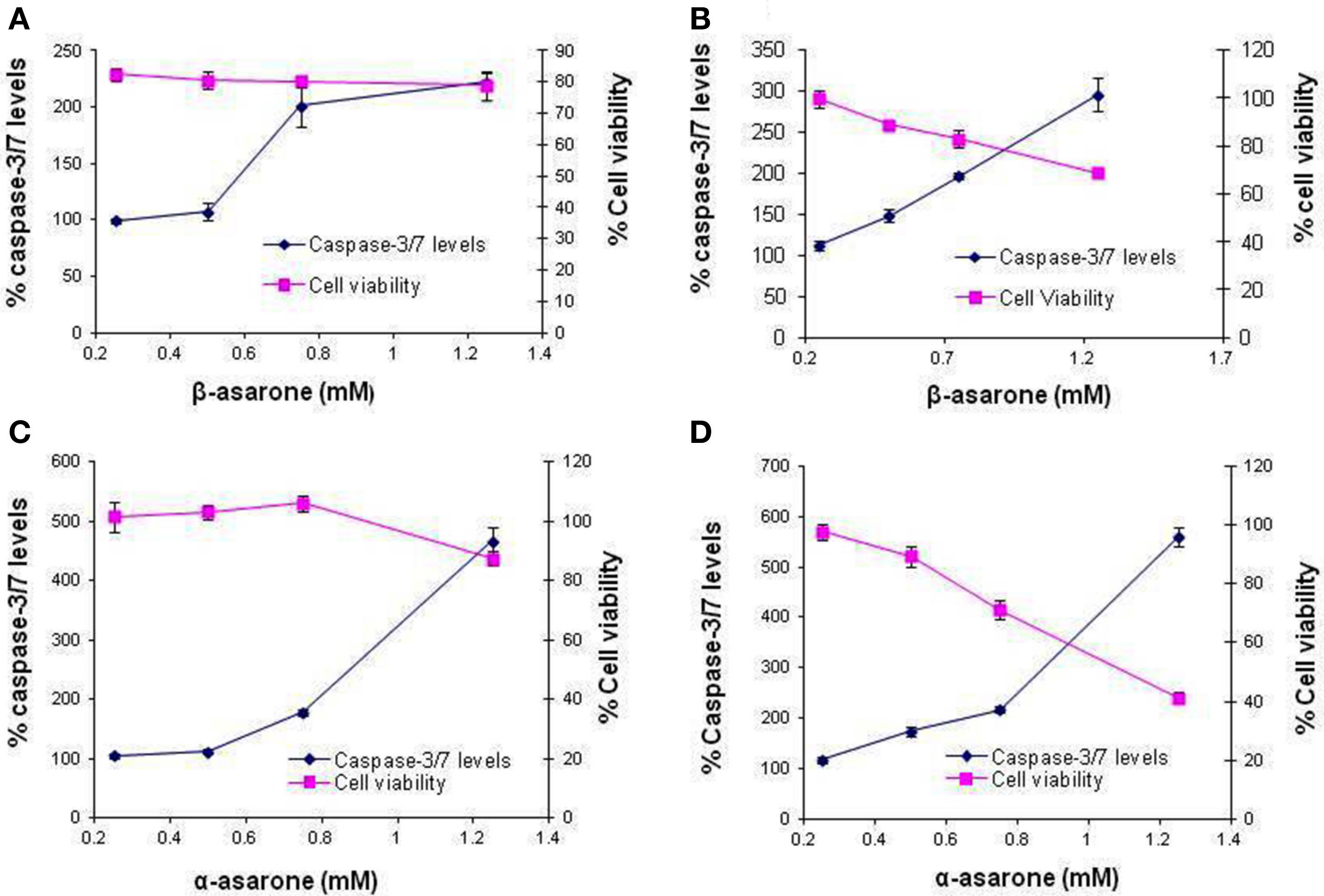
**Effects on caspase-3/7 activation and cell viability after treatment with (A) β-asarone for 6 h, (B) β-asarone for 12 h, (C) α-asarone for 6 h and (D) α-asarone for 12 h**. The plot represents average % caspase-3/7 levels on primary Y-axis and % cell viability on secondary Y-axis ± standard deviation at different concentrations.

Effects of α and β-asarone on caspase-3/7 levels were determined after incubating THLE-2 cells for 6 and 12 h. THLE-2 cells were exposed to four different concentrations of asarones to evaluate the dose dependent effects of asarones in casapse-3/7 levels. Increased levels of caspase-3/7 accompanied by reduced cell viability were observed upon treatment with asarones at both timepoints as compared to control cells not receiving treatment (Figure 5). This implies that α-asarone and β-asarone could cause cell death by apoptosis as evident by increased levels of caspase-3/7. Compared to β-asarone, the α-asarone was more potent in inducing caspase-3/7 levels. After 12 h of incubation with α-asarone, the levels of caspase-3/7 were 600% more than that of control cells.

## Discussion

Compared to the extensive investigations of hepatotoxicity due to prescription drugs, much less is known about the safety of herbal products (Willett et al., 2004). In some cases, hepatotoxicity has been related to the presence of undeclared prescription drugs and/or their synthetic analogs and ingestion of botanicals containing known hepatotoxins (e.g., pyrrolizidine alkaloids). Several reports of involvement of herbal products in drug-induced liver injury raise concerns on the safety of herbal products (Stickel et al., 2005; Navarro, 2009; Teschke et al., 2013). In our previous study involving analyses of adverse events reports associated with the use of herbal products in the Singapore Pharmacovigilance database over a 12 years period, we found that the liver was the major organ involved in serious adverse events (Patel et al., 2012). Hepatotoxicity associated with the use of herbal products and health supplements was responsible for 10 deaths in Singapore in the period 1998–2009 (Patel et al., 2012). There were 15 cases of hepatic failure in Singapore during 1998–2009. In one of those 15 cases, an herbal product (Product 20) from one batch was suspected to be implicated to cause acute liver failure in 3-month old baby. The suspected product was not found to be adulterated with any prescription drugs during routine screening in our laboratory and also by Health Sciences Authority, Singapore. However, high concentrations of β-asarone were detected in this product. Although asarones are potentially genotoxic and carcinogenic, their hepatotoxic effects have not been ascertained. Considering their detection in herbal products in children, and their potential toxic effects, it is important to estimate their presence in such products as well as to evaluate their hepatotoxic potential.

To achieve this, we developed and validated GC-MS method for the quantitative determination of asarones in herbal products. The advantage of our method is the short run-time of 12 min for simultaneous determination of asarones compared to previously reported GC-MS methods where minimum analysis time is 18 min. The developed method was successfully applied to screen for the presence of asarones in 20 herbal products. Some of the products were found to contain greater amounts of β-asarone than the recommended levels. In six out of ten products found to s contain asarones, the presence could be attributed to the presence of herbs containing asarones such as *Acori tatarinowii* Rhizoma*, Acori graminei* and *Asari radix e*t Rhizoma. In the remaining four samples found to contain asarones, the presence of asarones could not be linked to the labeled ingredients. Herbs *Acori tatarinowii* Rhizoma*, Acori graminei* and *Asari radix e*t Rhizoma belong to the same genus as *A. calamus*, a herb prohibited from use in any medicinal or food preparations in US The Electronic Code of Federal Regulations (e-CFR)^1^. To investigate the abundance of asarones in *Acori tatarinowii*, we have also quantified the concentration of β-asarone in this herb. According to the Chinese Pharmacopoeia, the recommended dose of this herb is 3–9 g (Chen et al., 2009). Based on such recommended dose in Chinese Pharmacopoeia and determined concentration in this study, the β-asarone exposure levels would be 23,000–69,000 μg, which is extremely higher than the recommended levels. The biological exposure and clinical significance are of course also highly dependent on the processing of the herb, bioavailability and intrinsic toxicity (if any) of the components. The limit of exposure from herbal medicinal products has been set at 115 μg β-asarone/day. To meet recommended exposure levels, the amount of this herb should not exceed 15 mg in daily dose of herbal products to ensure safe usage of this herb. This means that stricter quality control measures and regulatory actions would be required to monitor such a small amount of this herb in botanical health products. Unlike *A. calamus*, this herb is not controlled by any regulatory authorities for its concentration in herbal preparations and no limits have been set up except for Health Canada. The Canadian regulations do not allow this herb in food without prior licensing because of its potential health risks (Health Canada)^2^. The results of this study indicate a need to conduct detailed evaluation of this herb to consider its regulation in herbal preparations. Similarly, assuming the daily intake of the herbal drug to be 3 g for adults (Chen et al., 2009), the content of β-asarone per gram of herbal product should not exceed 38 μg per daily dose (Chen et al., 2009). If we apply this limit to the 10 products which were found to contain β-asarone, 8 of the products would exceed this limit. The results of this study highlight the importance of monitoring the levels of β-asarone in herbal products. Similar study involving determination of asarones content in herbal products for adults also reported high concentrations of asarones, especially for β-asarone (Zuba and Byearska, 2012). Herbal products in the form of pellets and tablets were seized by the police due to the intoxication of a young woman. Similar to our study, the concentrations of asarones in most of the examined pellets and tablets exceeded the maximum acceptable daily intake (Zuba and Byearska, 2012).

Chen et al. (2009), proposed extraction method involving preparation of decoction by heating to reduce the concentration of β-asarone in the *A, tatarinowii* extract. Their results indicated that by heating for 1 h, the concentration of β-asarone was reduced by 85%. However, even after this reduction, the β-asarone content was still very high (Chen et al., 2009). They proposed that extended heating for 1–3 h is required to achieve acceptable β-asarone levels. However, it is an unfavorable process for herbal preparations in practice. Moreover, in multi-component botanical health products, when this herb is added in powder form, the proposed extended heating method is not feasible. Therefore, further studies are required to derive practical and acceptable method of processing of this herb if it has to be used. Moreover, the product suspected to cause hepatotoxicity (Product 20) was not meant for its usage in infants even though it was given to the baby by parents. This suggests that there is a need to improve awareness amongst the consumer for correct use of botanical health products. Furthermore, label warnings such as “not for children's below one year” could also help to prevent such life-threatening adverse events. In addition, this study shows the importance of consulting a qualified practitioner before consuming any herbal products to avoid potential unwanted effects of herbal products. To the best of our knowledge, this is the first report of determination of asarones in herbal products for children.

To associate the observed high concentrations of asarones in the suspected product and other products purchased from the local herbal shops to their potential hepatotoxicity, we performed hepatotoxicity evaluation of asarones through various *in vitro* assays using immortalized human hepatocytes (THLE-2 cells) and investigated the possible mechanisms involved for the observed effects in hepatocytes. These immortalized human liver cells were selected for their metabolic competency and stability as an *in vitro* model for pharmacotoxicological studies (Pfeifer et al., 1993). THLE-2 cells are able to metabolize benzo[a]pyrene, N-nitrosodimethylamine, and aflatoxin B1 to their metabolites that adduct DNA, causing cancer. This indicates functionality similar to human liver (Pfeifer et al., 1993). THLE-2 cells have been used to study the hepatotoxicity of oral hypoglycaemic agents such as triglitazone (Saha et al., 2010a). Saha et al. (2010b) also reported that THLE-2 cells were able to metabolize triglitazone into its reactive metabolites. Furthermore, THLE-2 cells are also reported to be used at Pfizer, Astra Zeneca and Bristol-Mayer-Squibb for the screening of hepatotoxic potential of drug candidates (Greene et al., 2010; Thompson et al., 2011). In addition, THLE assay can also be used as a high throughput assay to prioritize compound selection for *in vivo* toxicity screening (Greene et al., 2010). THLE-2 cells have also been used as control cells representing normal human liver cells (Mayoral et al., 2005; Datta et al., 2007; Krajka-KuŸniak et al., 2012; Zimmerman et al., 2012).

The IC_50_ values obtained from the cell viability assay indicate that asarones and the methanol extract of Product 20 are cytotoxic to hepatocytes. Although β-asarone was found to be slightly more cytotoxic than α-asarone, the difference was not statistically significant. Pharmacokinetic study of β-asarone by Fang et al. (2012) to study the transformation and excretion of β-asarone in rabbits has shown that about 22% of β-asarone is converted to α-asarone in rabbits. Previous studies conducted on rat hepatocytes have identified trans-trimethoxycinnamic acid (trans-TMC) as the major metabolite of α-asarone. Trans-TMC was also identified as metabolites of asarones in *C. elegans*, microbial model for mammalian drug metabolism (Björnstad et al., 2009). In contrast, in humans, cis-TMC and hydroxylated β-asarone were identified as major metabolites of asarones in an analysis of urine samples of patients with *A. calamus* oil intoxications in Sweden (Björnstad et al., 2009). As trans-TMC is commercially available, further experiments were conducted to determine the toxicity of trans-TMC in hepatocytes. However, trans-TMC was found to be five times less toxic than the β-asarone. Therefore, the trans-TMC does not seem to be the toxic metabolite of asarones. Our results and the study by Björnstad et al. (2009) suggests that metabolites other than trans-TMC, such as cis- TMC could be involved in the toxicity of asarones. Cis-TMC is not commercially available and its toxicity could not be assessed in the present study.

To investigate possible mechanisms of the cytotoxicity in hepatocytes, we determine lipid peroxidation and glutathione depletion in THLE-2 cells after treatment with asarones, as biochemical indicators of oxidative stress (Tripathi et al., 2009). Elevation of ROS in hepatocytes is reported to be one of the main mechanisms for drug induced hepatotoxicity. This ROS induced hepatic cell toxicity leads to a variety of manifestations of liver diseases such as ischemia–reperfusion injury, fibrosis and liver failure (Novo and Parola, 2008). These outcomes arise from damaging effect of ROS on various subcellular components. For example, these radicals cause peroxidation of the cell membrane phospholipids which results in accumulation of lipid peroxides leading to the disruption of membrane structure and function. ROS also affects the anti-oxidant defense system involving glutathione. Glutathione (GSH) is an abundant antioxidant, which protects cells from ROS such as free radicals and peroxides (Griffith, 1999; Sies, 1999) through sacrificial oxidation to its oxidized form glutathione disulfide (GSSG) during oxidative stress. In healthy cells and tissue, more than 90% of the total glutathione pool is in the reduced form (GSH) and less than 10% exists in the oxidized form (GSSG). A reduced ratio of GSH to GSSG within cells is used as an indicator of oxidative stress and cellular toxicity (Ghezzi, 2005; Ballatori et al., 2009). The depletion of total GSH also suggests adduct formation with reactive metabolites (in the form of GSH conjugates).

From lipid peroxidation assay, α and β-asarones and Product 20 were found to cause oxidative stress in a dose-dependent manner as indicated by the increased formation of MDA. Similar observations were made with glutathione depletion assay. This data suggests that pure asarones and products containing high concentrations of asarones are producing cellular damage in hepatocytes by oxidative stress. While the exact chemical entity responsible for the damage is not directly investigated, previous studies pointed at the formation of reactive metabolites which are capable of generating ROS. Hasheminejad and Caldwell (1994) reported that genotoxicity of asarones could be due to their oxidized metabolites such as trans-2, 4, 5-trimethoxycinnamic acid which is the product of ω-oxidation. Their conclusion was based on the observation that the CYP-450 inhibitor, cimetidine, was able to block the genotoxicity of asarones (Hasheminejad and Caldwell, 1994). However, trans-2, 4, 5-trimethoxycinnamic acid exhibited little toxicity in our cell model as compared to the parental asarones. Other reactive metabolites could arise from epoxidation of carbon-carbon double bond of asarones (Hasheminejad and Caldwell, 1994). Moreover, the w-oxidation and subsequent sulfation of asarones could also lead to formation of reactive carbonium ions (Tsai et al., 1994). Therefore, further work is required to characterize the metabolites responsible for the observed toxicity of asarones.

After investigating the oxidative stress inducing effects of asarones in hepatocytes, we characterized the mode of cell death that ensued this injury. Apoptosis is usually manifested by activation of specific proteases such as caspase-3 and 7. α-asarone and β-asarone were found to induce caspase-3/7 activation implying cell death by apoptosis. These results corroborated earlier works where β-asarone directly induced apoptosis in LoVo colon cancer cells by up-regulation of caspases through a mitochondrial pathway *in vitro* and *in vivo* (Zou et al., 2012). However, β-asarone is also reported to reduce apoptosis by inhibiting caspase-3 activation (Li et al., 2010; Liu et al., 2012). However, direct comparison cannot be made as their study involved investigations of the effect of β-asarone after deliberately inducing apoptosis by use of neurotoxin and also there are differences in cellular system used. This comparison further accentuates the importance of using tissue-specific system to derive mechanistic information of relevance to clinical observations, such as in herb-mediated hepatotoxicity. To the best of our knowledge, this is the first report of the effects of asarones on human liver cells. The presence of high concentration of potentially toxic asarones in herbal products and their unsupervised consumption especially in infants and young children who are more prone to hepatotoxicity sets the impetus to understand more about the role of asarones in *in vivo* drug induced liver injury.

## Conclusion

In this study, a simple, rapid and validated GC-MS method for quantitative determination of asarones in herbal products is developed and applied for the analysis of herbal products used in children including a product suspected to be implicated in hepatotoxicity. In addition, asarones were also evaluated for their hepatotoxic effects using human hepatocytes and possible mechanisms of their observed effects were also studied. High concentrations of asarones were found in some of the tested products. Asarones were also found to exhibit cytotoxicity to hepatocytes possibly by inducing oxidative stress. The results of this study shed light on the importance of controlling the concentration of potentially toxic asarones in herbal products to safeguard consumer safety especially young children. Further investigations of the toxicity of asarones are warranted.

### Conflict of interest statement

The authors declare that the research was conducted in the absence of any commercial or financial relationships that could be construed as a potential conflict of interest.

## Footnotes

^1^The Electronic Code of Federal Regulations (e-CFR), Title 21, Chapter I, Subchapter B, Part 189, Available online at: http://www.ecfr.gov/cgi-bin/text-idx?c=ecfr&rgn=div5&view=text&node=21:3.0.1.1.16&idno=21#se21.3.189_1110 (Accessed November 12, 2014).

^2^Natural Health Products Ingredients Database. Available online at: http://webprod.hc-sc.gc.ca/nhpid-bdipsn/ingredReq.do?id=13339&lang=eng (Accessed February 13, 2015).

## References

[B1] Abdualmjid R. J. Sergi C. (2013). Hepatotoxic botanicals—an evidence-based systematic review. J. Pharm. Pharm. Sci. 16, 376–404. 24021288 10.18433/j36g6x

[B2] Ballatori N. Krance S. M. Notenboom S. Shi S. Tieu K. Hammond C. L. (2009). Glutathione dysregulation and the etiology and progression of human diseases. Biol. Chem. 390, 191–214. 10.1515/BC.2009.033 19166318 PMC2756154

[B3] Björnstad K. Helander A. Hultén P. Beck O. (2009). Bioanalytical investigation of asarone in connection with *Acorus calamus* oil intoxications. J. Anal. Toxicol. 33, 604–609. 10.1093/jat/33.9.604 20040135

[B4a] Chen C. Spriano D. Meier B. (2009). Reduction of beta-asarone in acori rhizoma by decoction. Planta Med. 75, 1448–1452. 10.1055/s-0029-1185742 19507115

[B4] Chen H. Li W. G. Zhang X. B. Wang L. Xu T. L. Wu D. Z.. (2013). Alpha-asarone from *Acorus gramineus* alleviates epilepsy by modulating A-type gaba receptors. Neuropharmacology 65, 1–11. 10.1016/j.neuropharm.2012.09.001 22975146

[B5] Cho J. Kim Y. H. Kong J. Y. Yang C. H. Park C. G. (2002). Protection of cultured rat cortical neurons from excitotoxicity by asarone, a major essential oil component in the rhizomes of *Acorus gramineus*. Life Sci. 71, 591–599. 10.1016/S0024-3205(02)01729-0 12052443

[B6] Commission of Chinese Pharmacopoeia (2005). Pharmacopoeia of the People's Republic of China, Vol. 1 Beijing: China Medico-Pharmaceutical Science and Technology Publishing House.

[B7] Datta J. Majumder S. Kutay H. Motiwala T. Frankel W. Costa R.. (2007). Metallothionein expression is suppressed in primary human hepatocellular carcinomas and is mediated through inactivation of CCAAT/enhancer binding protein α by phosphatidylinositol 3-kinase signalling cascade. Cancer Res. 67, 2736–2746. 10.1158/0008-5472.CAN-06-4433 17363595 PMC2276570

[B8] European Medicines Agency (EMEA) (2002). Scientific Committee on Food, European Commission. Opinion of the Scientific Committee on Food on the Presence of β-asarone in Flavorings and Other Food Ingredients with Flavoring Properties (SCF/CS/FLAV/FLAVOUR/9 ADD1 Final), Brussels.

[B9] European Medicines Agency (EMEA) (2005). Committee on Herbal Medicinal Products, Evaluation of Medicines for Human Use, Public Statement on the Use of Herbal Medicinal Products Containing Asarone (Doc Ref: EMEA/HMPC/139215/2005), London.

[B10] Fang Y. Q. Shi C. Liu L. Fang R. M. (2012). Analysis of transformation and excretion of β-asarone in rabbits with GC-MS. Eur. J. Drug Metab. Pharmacokinet. 37, 187–190. 10.1007/s13318-012-0083-z 22351074

[B10a] Fu S. Y. Fang R. M. Fang G. L. Xie Y. H. Fang Y. Q. (2008). Effects of beta-asarone on expression of FOS and GAD65 in cortex of epileptic rat induced by penicillin. Zhong Yao Cai. 31, 79–81. 18589755

[B11] Garduno L. Salazar M. Salazar S. Morelos M. E. Labarrios F. Tamariz J. A.. (1997). Hypolipidaemic activity of a-asarone in mice. J. Ethnopharmacol. 55, 161–163. 10.1016/S0378-8741(96)01492-4 9032629

[B12] Geng Y. Li C. Liu J. Xing G. Zhou L. Dong M.. (2010). Beta-asarone improves cognitive function by suppressing neuronal apoptosis in the beta amyloid hippocampus injection rats. Biol. Pharm. Bull. 33, 836–843. 10.1248/bpb.33.836 20460763

[B13] Ghezzi P. (2005). Regulation of protein function by glutathionylation. Free Radic. Res. 39, 573–580. 10.1080/10715760500072172 16036334

[B14] Greene N. Aleo M. D. Louise-May S. Price D. A. Will Y. (2010). Using an *in vitro* cytotoxicity assay to aid in compound selection for *in vivo* safety studies. Bioorg. Med. Chem. Lett. 20, 5308–5312. 10.1016/j.bmcl.2010.06.129 20655216

[B15] Griffith O. W. (1999). Biologic and pharmacologic regulation of mammalian glutathione synthesis. Free Radic. Biol. Med. 27, 922–935. 10.1016/S0891-5849(99)00176-8 10569625

[B16] Hanson K. M. Gayton-Ely M. Holland L. A. Zehr P. S. Söderberg B. C. (2005). Rapid assessment of β-asarone content of *Acorus calamus* by micellar electrokinetic capillary chromatography. Electrophoresis 26, 943–946. 10.1002/elps.200410165 15714542

[B17] Hasheminejad G. Caldwell J. (1994). Genotoxity of the alkenylbenzenes α-asarone and β-asarone, myristicin and elemicin as determined by the UDS assay in cultured rat hepatocytes. Food Chem. Toxicol. 32, 223–231. 8157216 10.1016/0278-6915(94)90194-5

[B56] Kaplowitz N. (1997). Hepatotoxicity of herbal remedies: insights into the intricacies of plant-animal warfare and cell death. Gastroenterology 113, 1408–1412. 10.1053/gast.1997.v113.agast971131408 9322538

[B18] Krajka-KuŸniak V. Paluszczak J. Celewicz L. Barciszewski J. Baer-Dubowska W. (2012). Phloretamide, an apple phenolic compound, activates the Nrf2/ARE pathway in human hepatocytes. Food Chem. Toxicol. 51C, 202–209. 10.1016/j.fct.2012.09.033 23063670

[B19] Kumari R. Agrawal S. B. Singh S. Dubey N. K. (2009). Supplemental ultraviolet-B induced changes in essential oil composition and total phenolics of *Acorus calamus* L. (sweet flag). Ecotoxicol. Environ. Saf. 72, 2013-2019. 10.1016/j.ecoenv.2009.02.006 19321203

[B20] Li C. C. Xing G. H. Dong M. X. Zhou L. Li J. M. Wang G. Zou D. J.. (2010). β-asarone protection against β-amyloid-induced neurotoxicity in PC12 cells via JNK signal modulation of Bcl-2 family proteins. Eur. J. Pharmacol. 635, 96–102. 10.1016/j.ejphar.2010.03.013 20307525

[B21] Li Z. Zhao G. Qian S. Yang Z. Chen X. Chen J.. (2012). Cerebrovascular protection of beta-asarone in Alzheimer's disease rats: a behavioral, cerebral blood flow, biochemical and genic study. J. Ethnopharmacol. 144, 305–312. 10.1016/j.jep.2012.09.013 22985635

[B22] Lim H. W. Kumar H. Kim B. W. More S. V. Kim I. W. Park J. I.. (2014). β-asarone (cis-2,4, 5-trimethoxy-1-allyl phenyl), attenuates pro inflammatory mediators by inhibiting NF-κB signaling and the JNK pathway in LPS activated BV-2 microglia cells. Food Chem. Toxicol. 72, 265–272. 10.1016/j.fct.2014.07.018 25066769

[B23] Limón I. D. Mendieta L. Díaz A. Chamorro G. Espinosa B. Zenteno E.. (2009). Neuroprotective effect of α-asarone on spatial memory and nitric oxide levels in rats injected with amyloid-β. Neurosci. Lett. 453, 98–103. 10.1016/j.neulet.2009.02.011 19356601

[B24] Liu J. Li C. Xing G. Zhou L. Dong M. Geng Y.. (2010). Beta-asarone attenuates neuronal apoptosis induced by beta amyloid in rat hippocampus. Yakugaku Zasshi 130, 737–746. 10.1248/yakushi.130.737 20460873

[B25] Liu L. Fang Y. Q. Xue Z. F. He Y. P. Fang R. M. Li L. (2012). β-asarone attenuates ischemia reperfusion-induced autophagy in rat brains via modulating JNK, p-JNK, Bcl-2 and Beclin 1. Eur. J. Pharmacol. 680, 34–40. 10.1016/j.ejphar.2012.01.016 22306244

[B25a] López M. L. Hernàndez A. Chamorro G. Mendoza-Figueroa T. (1993). alpha-Asarone toxicity in long-term cultures of adult rat hepatocytes. Planta Med. 59, 115–120. 10.1055/s-2006-959624 8488189

[B26] MacGregor F. B. Abernethy V. E. Dahabra S. Cobden I. Hayes P. C. (1989). Hepatotoxicity of herbal remedies. BMJ 299:1156. 10.1136/bmj.299.6708.1156 2513032 PMC1838039

[B27] Mayoral R. Fernández-Martínez A. Boscá L. Martín-Sanz P. (2005). Prostaglandin E2 promotes migration and adhesion in hepatocellular carcinoma cells. Carcinogenesis 26, 753–761. 10.1093/carcin/bgi022 15661807

[B28] Meng X. Liao S. Wang X. Wang S. Zhao X. Jia P.. (2014). Reversing P-glycoprotein-mediated multidrug resistance *in vitro* by α-asarone and β-asarone, bioactive cis-trans isomers from *Acorus tatarinowii*. Biotechnol. Lett. 36, 685–691. 10.1007/s10529-013-1419-8 24322772

[B29] Nandakumar S. Menon S. Shailajan S. (2013). A rapid HPLC-ESI-MS/MS method for determination of β-asarone, a potential anti-epileptic agent, in plasma after oral administration of *Acorus calamus* extracts on rats. Biomed. Chromatogr. 27, 318–326. 10.1002/bmc.2794 22903588

[B30] Navarro V. J. (2009). Herbal and dietary supplement hepatotoxicity. Semin. Liver Dis. 29, 373–382. 10.1055/s-0029-1240006 19826971

[B31] Navarro V. J. Barnhart H. Bonkovsky H. L. Davern T. Fontana R. J. Grant L.. (2014). Liver injury from herbals and dietary supplements in the U.S. Drug-Induced Liver Injury Network. Hepatology 60, 1399–1408. 10.1002/hep.27317 25043597 PMC4293199

[B32] Navarro V. J. Lucena M. I. (2014). Hepatotoxicity induced by herbal and dietary supplements. Semin. Liver Dis. 34, 172–193. 10.1055/s-0034-1375958 24879982

[B33] Novo E. Parola M. (2008). Redox mechanisms in hepatic chronic wound healing and fibrogenesis. Fibrogenesis Tissue Repair 1:5 10.1186/1755-1536-1-5 19014652 PMC2584013

[B34] Oprean R. Tamas M. Roman L. (1998). Comparison of GC-MS and TLC techniques for asarone isomers determination. J. Pharm. Biomed. Anal. 18, 227–234. 10.1016/S0731-7085(98)00161-7 9863962

[B35] Patel D. N. Low W. L. Tan L. L. Tan M. M. Zhang Q. Low M. Y.. (2012). Adverse events associated with the use of complementary medicine and health supplements: an analysis of reports in the Singapore Pharmacovigilance database from 1998 to 2009. Clin. Toxicol. (Phila). 50, 481–489. 10.3109/15563650.2012.700402 22738039

[B36] Pfeifer A. M. Cole K. E. Smoot D. T. Weston A. Groopman J. D. Shields P. G.. (1993). Simian virus 40 large tumor antigen-immortalized normal human liver epithelial cells express hepatocyte characteristics and metabolize chemical carcinogens. Proc. Natl. Acad. Sci. U.S.A. 90, 5123–5127. 10.1073/pnas.90.11.5123 7685115 PMC46667

[B37] Saha S. New L. S. Ho H. K. Chui W. K. Chan E. C (2010a). Direct toxicity effects of sulfo-conjugated troglitazone on human hepatocytes. Toxicol. Lett. 195, 135–141. 10.1016/j.toxlet.2010.03.010 20307632

[B38] Saha S. New L. S. Ho H. K. Chui W. K. Chan E. C. (2010b). Investigation of the role of the thiazolidinedione ring of troglitazone in inducing hepatotoxicity. Toxicol. Lett. 192, 141–149. 10.1016/j.toxlet.2009.10.014 19854250

[B39] Sandeep D. Nair C. K. (2011). Radioprotection by α-asarone: prevention of genotoxicity and hematopoietic injury in mammalian organism. Mutat. Res. 722, 62–68. 10.1016/j.mrgentox.2011.03.005 21440084

[B40] Sies H. (1999). Glutathione and its role in cellular functions. Free Radic. Biol. Med. 27, 916–921. 10.1016/S0891-5849(99)00177-X 10569624

[B40a] Strader D. B. Navarro V. J. Seeff L. B. (2012). Chapter 26 - Hepatotoxicity of herbal preparations, in Zakim and Boyer's Hepatology, 6th Edn, eds Boyer T. D. Manns M. P. Sanyal A. J. (Saint Louis, MO: W. B. Saunders), 462–475 10.1016/B978-1-4377-0881-3.00026-7

[B41] Stedman C. (2002). Herbal hepatotoxicity. Semin. Liver. Dis. 22, 195–206. 10.1055/s-2002-30104 12016550

[B42] Stickel F. Patsenker E. Schuppan D. (2005). Herbal hepatotoxicity. J. Hepatol. 43, 901–910 10.1016/j.jhep.2005.08.002 16171893

[B43] Teschke R. Frenzel C. Glass X. Schulze J. Eickhoff A. (2013). Herbal hepatotoxicity: a critical review. Br. J. Clin. Pharmacol. 75, 630–636. 10.1111/j.1365-2125.2012.04395.x 22831551 PMC3575930

[B43a] Thompson R. A. Isin E. M. Li Y. Weaver R. Weidolf L. Wilson I.. (2011). Risk assessment and mitigation strategies for reactive metabolites in drug discovery and development. Chem. Biol. Interact. 192, 65–71. 10.1016/j.cbi.2010.11.002 21074519

[B44] Tripathi M. Singh B. K. Kakkar P. (2009). Glycyrrhizic acid modulates t-BHP induced apoptosis in primary rat hepatocytes. Food Chem. Toxicol. 47, 339–347. 10.1016/j.fct.2008.11.028 19084568

[B45] Tsai R. S. Carrupt P. A. Testa B. Caldwell J. (1994). Structure-genotoxicity relationships of allylbenzenes and propenylbenzenes: a quantum chemical study. Chem. Res. Toxicol. 7, 73–76. 10.1021/tx00037a011 8155828

[B46] Unger P. Melzig M. F. (2012). Comparative study of the cytotoxicity and genotoxicity of alpha- and beta-asarone. Sci. Pharm. 80, 663–668. 10.3797/scipharm.1204-21 23008813 PMC3447612

[B47] Wang Z. Wang Q. Yang B. Li J. Yang C. Meng Y.. (2014). GC-MS method for determination and pharmacokinetic study of four phenylpropanoids in rat plasma after oral administration of the essential oil of *Acorus tatarinowii* Schott rhizomes. J. Ethnopharmacol. 155, 1134–1140. 10.1016/j.jep.2014.06.035 25046827

[B48] Wei G. Chen Y.-B. Chen D.-F. Lai X.-P. Liu D.-H. Deng R.-D.. (2013). β-asarone inhibits neuronal apoptosis via the CaMKII/CREB/Bcl-2 signaling pathway in an *in vitro* model and AbPP/PS1 mice. J. Alzheimer's Dis. 33, 863–880. 10.3233/JAD-2012-120865 23064259

[B49] Widmer V. Schibli A. Reich E. (2005). Quantitative determination of β-asarone in calamus by high-performance thin-layer chromatography. J. AOAC Int. 88, 1562–1567. 16386010

[B50] Willett K. L. Roth R. A. Walker L. (2004). Workshop overview: hepatotoxicity assessment for botanical dietary supplements. Toxicol. Sci. 79, 4–9. 10.1093/toxsci/kfh075 14976355

[B51] Wu Q. D. Fang Y. Q. Chen Y. Z. Kuan Z. S. Wang S. Y. He Y. P. (2005). Protective effects of volatile oil of *Acorus tatarinowii* Schott and β-asarone on cardiovascular system. Tradit Chin. Drug Res. Clin. Pharmacol. 4, 244–247.

[B51a] Yang Y. X. Chen Y. T. Zhou X. J. Hong C. L. Li C. Y. Guo J. Y. (2013). Beta-asarone, a major component of Acorus tatarinowii Schott, attenuates focal cerebral ischemia induced by middle cerebral artery occlusion in rats. BMC Complement. Altern. Med. 13:236. 10.1186/1472-6882-13-236 24066702 PMC3853232

[B52] Zimmerman J. W. Pennison M. J. Brezovich I. Yi N. Yang C. T. Ramaker R.. (2012). Cancer cell proliferation is inhibited by specific modulation frequencies. Br. J. Cancer 106, 307–313. 10.1038/bjc.2011.523 22134506 PMC3261663

[B53] Zou D. J. Wang G. Liu J. C. Dong M. X. Li X. M. Zhang C.. (2011). β-asarone attenuates β-amyloid-induced apoptosis through the inhibition of the activation of apoptosis signal-regulating kinase1 in SH-SY5Y cells. Pharmazie 66, 44–51. 21391434

[B54] Zou X. Liu S. L. Zhou J. Y. Wu J. Ling B. F. Wang R. P. (2012). Beta-asarone induces LoVo colon cancer cell apoptosis by up-regulation of caspases through a mitochondrial pathway *in vitro* and *in vivo*. Asian Pac. J. Cancer Prev. 13, 5291–5298. 10.7314/APJCP.2012.13.10.5291 23244151

[B55] Zuba D. Byearska B. (2012). Alpha- and beta-asarone in herbal medicinal products. Forensic Sci. Int. 223, e5–e9. 10.1016/j.forsciint.2012.08.015 22964166

