# Supplementary material for: Hepatotoxic potential of asarones: in vitro evaluation of hepatotoxicity and quantitative determination in herbal products
Source: Front Pharmacol. 2015 Feb 20;6:25. doi: 10.3389/fphar.2015.00025 (PMC4335289; doi:10.3389/fphar.2015.00025)
Supplement: Supplementary file 1 [file Table1.DOC]

**SUPPLEMENTARY MATERIAL**

**Hepatotoxic potential of asarones: *In vitro* evaluation of hepatotoxicity and quantitative determination in herbal products**

Dhavalkumar Narendrabhai Patel^1^, Han Kiat Ho^1^*, Libei Liesbet Tan^2^, Mui-Mui Belinda Tan^2^, Qian Zhang ^2^, Min-Yong Low^3^, Cheng-Leng Chan^2^, Hwee-Ling Koh^1^*

1. Department of Pharmacy, Faculty of Science, National University of Singapore, 18 Science Drive 4, Singapore 117543

2. Health Products Regulation, Vigilance, Compliance and Enforcement Division, Health Sciences Authority, Singapore

3. Applied Sciences Group, Pharmaceutical Division, Health Sciences Authority, Singapore

*** Correspondence:**

Associate Professor Hwee-Ling Koh, Department of Pharmacy, Faculty of Science, National University of Singapore, 18 Science Drive 4, Singapore 117543,Tel: (65) 65167962, Fax: (65) 67791554, Email: [phakohhl@nus.edu.sg](mailto:phakohhl@nus.edu.sg)

Assistant Professor Han Kiat Ho, Department of Pharmacy, Faculty of Science, National University of Singapore, 18 Science Drive 4, Singapore 117543,Tel: (65) 65167963, Fax: (65) 67791554, Email: [phahohk@nus.edu.sg](mailto:phahohk@nus.edu.sg)

**Table 1**  Analytical recoveries of asarones

| **No.** | **Asarone** | **Concentration**  **Spiked (mg/g)** | **Mean recovery (n=3)** | |
| --- | --- | --- | --- | --- |
|  |  |  | **% recovery ± SD** | **% RSD** |
| 1 | β-asarone | 0.060 | 96.40 ± 4.94 | 5.13 |
|  |  | 0.150 | 99.58 ± 2.83 | 2.84 |
|  |  | 0.600 | 96.33 ± 6.50 | 6.74 |
| 2 | α-asarone | 0.060 | 100.08 ± 2.74 | 2.73 |
|  |  | 0.150 | 101.74 ± 5.48 | 5.38 |
|  |  | 0.600 | 97.83 ± 3.61 | 3.69 |

**Table 2** Stability of α and β-asarone under various conditions

| **No.** | **Asarone** | **QC sample (ng/mL)** | **Stability of stock solution after one week at 24 ^o^C (% remained ± SD), n = 3** | **Post preparation stability after 48 hours 24 ^o^C (% remained ± SD),**  **n = 3** |
| --- | --- | --- | --- | --- |
| 1 | β-asarone | 400 | 95.35 ± 2.40 | 95.80 ± 4.09 |
|  |  | 1000 | 96.24 ± 6.27 | 100.13 ± 1.33 |
|  |  | 4000 | 99.56 ± 0.10 | 99.20 ± 3.41 |
| 2 | α-asarone | 400 | 95.65 ± 2.32 | 91.50 ± 1.73 |
|  |  | 1000 | 93.64 ± 6.05 | 93.60 ± 4.38 |
|  |  | 4000 | 98.94 ± 1.83 | 101.74 ± 5.48 |
